# Evaluation of Pain in Infants of the First 100 Days of Age With Artificial Intelligence Techniques

**DOI:** 10.1111/jep.70497

**Published:** 2026-06-16

**Authors:** Ayfer Acikgoz, Deniz Yigit, Ozer Celik

**Affiliations:** ^1^ Department of Child Health and Diseases Nursing, Faculty of Health Sciences Eskisehir Osmangazi University Eskişehir Turkey; ^2^ Department of Child Health and Diseases Nursing, Faculty of Health Sciences Kütahya Health Sciences University Kütahya Turkey; ^3^ Artificial Intelligence Application and Research Center Anadolu University Eskişehir Turkey

**Keywords:** artificial intelligence, neonatal intensive care unit, newborn, N‐PASS, nurse, pain

## Abstract

**Rationale:**

Pain assessment in infants is difficult due to the lack of verbal communication and the subjective nature of existing methods. Current tools require evaluating multiple indicators separately, which can be time‐consuming and variable between observers. Artificial intelligence can enable rapid and standardised direct pain scoring, improving accuracy and clinical efficiency. The limited number of comprehensive studies in this area highlights an important gap in the literature.

**Aims and Objectives:**

This research was conducted to develop an application that will evaluate pain in infants in the first 100 days of life using artificial intelligence techniques.

**Methods:**

This study is an artificial intelligence–based research conducted with 1000 newborns hospitalised in a Neonatal Intensive Care Unit. A data collection form and the Neonatal Pain, Agitation, and Sedation Scale were used as data collection tools. Data analysis was performed using IBM SPSS Statistics 21.0, with descriptive statistics and Cohen's Kappa test applied. A *p* value of < 0.05 was considered statistically significant. Image and audio recordings from all 1000 newborns were independently labelled by the researchers, and these labelled data were used to develop the artificial intelligence model. The labelled image and audio recordings were utilised for model training (80%), validation (10%), and testing (10%).

**Results:**

The mean gestational age of infants was determined to be 38.52 ± 1.07 weeks, and the postnatal age was determined to be 2.90 ± 0.77 days. It was determined that there was no difference between the pain scale scores labelled by the researchers (*p* > 0.05). It was determined that the success rate of the created artificial intelligence model in correctly predicting the presence of pain in newborns was 82%.

**Conclusions:**

It was determined that the developed artificial intelligence model was successful in predicting the presence of pain in newborns in the Neonatal Intensive Care Unit.

## 1 Introduction

From birth, infants are exposed to painful procedures such as heel lance, venipuncture, and vaccination, particularly in Neonatal Intensive Care Units [1]. These procedures have short‐ and long‐term adverse effects. Short‐term effects include physiological instability (e.g., increased heart rate, blood pressure, respiratory changes, decreased oxygen saturation), behavioural responses (crying, grimacing, restlessness), and sleep disturbances [2, 3]. Repeated pain exposure in the long term may affect brain structure and lead to behavioural and physical problems, including altered pain perception and reduced pain tolerance [4, 5, 6, 7]. Therefore, accurate pain assessment and timely intervention from birth are essential [3].

Pain assessment is straightforward in verbally communicative individuals but remains challenging in infants. Although various pain assessment scales have been developed, heavy workloads, multiple scale options, and paper‐based assessments limit their routine clinical use [2, 8]. Thus, more practical and efficient approaches are needed. Artificial intelligence (AI) is a promising solution, as AI‐supported systems may enable continuous and more effective pain assessment in infants [9, 10]. Although AI research in healthcare has increased, it has largely focused on physician–engineer collaborations, with nurses being underrepresented despite their central role in continuous patient care and pain management [9, 11, 12]. AI‐based systems that generate integrated pain scores may reduce nurses' workload and improve clinical efficiency. To our knowledge, this approach has not been previously studied. Therefore, this study aimed to evaluate pain in infants during the first 100 days of life using AI techniques. This study was conducted to evaluate pain in infants in the first 100 days with AI technique.

## 2 Methods

### 2.1 Type of Research

The research is a study of AI.

### 2.2 Place and Time of the Research

The study data were collected in the NICU of a hospital in Turkey between 15.01.2021 and 15.06.2024.

### 2.3 Universe and Sample Characteristics

**Figure 1:**
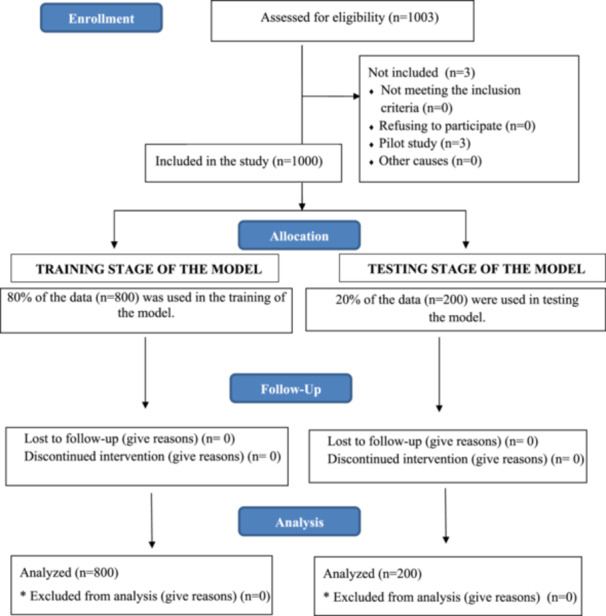
CONSORT 2010 flow diagram. CONSORT, consolidated standards of reporting trials.

Power analysis indicated that a sample size of *n* = 45 was sufficient to detect infant pain levels with an effect size of 0.50, an *α* of 0.05, and 87% power [13]. However, given the need for large datasets in AI research [14], the study was completed with 1000 infants. Post hoc power analysis showed 100% power with the same effect size and *α* level, satisfying both statistical adequacy and AI model development requirements. The study followed the CONSORT 2010 flow diagram (Figure 1). Infants were included if parental consent was obtained, they were term, and aged 0–100 days. Exclusion criteria included age over 100 days, receipt of analgesic and/or sedative medication within the previous 24 h, and conditions limiting pain assessment, such as intracranial haemorrhage or neuromotor developmental delay.

### 2.4 Ethical Aspects of the Research

Prior to the initiation of the study, permission to use the scale and ethical approval were obtained from the Eskişehir Osmangazi University Ethics Committee (09.05.2019; Decision No: 08) and from the relevant hospital administration (05.10.2020). Parents were informed about the study in advance, and both written and verbal informed consent were obtained. Only newborns whose parents provided consent were included in the study procedures.

### 2.5 Data Collection Tools

#### 2.5.1 Data Collection Form

The data collection form was developed by the researcher based on a review of the literature [3, 15, 16, 17, 18]. The form consisted of 10 questions addressing the infant's gestational week and postnatal age, sex, mode of delivery, feeding method, time of last feeding, use of analgesic/sedative medication, history of surgical intervention, and existing health problems. The form was completed by the researcher in collaboration with the mother and/or father, who agreed to participate in the study.

#### 2.5.2 Neonatal Pain, Agitation and Sedation Scale (N‐PASS)

The N‐PASS assesses pain in term and preterm infants during the first 100 days of life and was developed by Hummel et al. and adapted into Turkish by Acikgoz et al. [15, 16, 17, 18]. Cronbach's *α* was 0.797 before and 0.917 during/after the procedure. N‐PASS includes pain and sedation dimensions. The pain dimension evaluates five parameters scored from 0 to +2, yielding total scores from 0 to +11, with ≤3 indicating adequate pain control. In this study, only infants over 30 gestational weeks were included, and only the pain dimension was used [15, 16, 17, 18].

### 2.6 Data Collection

A pilot application was conducted with three infants who were not included in the study sample, confirming that camera placement in front of the incubator allowed adequate visualisation of all N‐PASS parameters and that 1‐min recordings per infant were sufficient for AI‐based analysis. Each newborn was then recorded once for 1 min at non‐fixed time points, and recordings from infants with and without pain were included to represent all pain scores (*n* = 1000). From each recording, a 3–5 s segment was labelled to assess N‐PASS parameters, and one pain score was calculated per infant. Pain scores were independently evaluated by three researchers, demonstrating high inter‐rater agreement (*p* < 0.001), and recordings assessed by one researcher were used for model development. The AI model and mobile application were developed by the service provider, with 80% of the data used for training, 10% for validation, and 10% for testing. Image‐based pain prediction was performed using pose estimation and feature extraction with CNN and LSTM architectures; audio data were analysed using LSTM models, and final predictions were obtained by combining image and audio outputs. A cross‐platform mobile application supporting iOS and Android was developed using the Flutter SDK to enable video upload, pain assessment, and storage of results.

### 2.7 Data Evaluation

Statistical analyses were performed using IBM SPSS Statistics 21.0 (IBM, 2021), and power analysis was conducted with PASS 2007. Descriptive statistics were used to summarise newborn characteristics. Inter‐rater agreement for N‐PASS scores was evaluated using Cohen's Kappa test, with *p* < 0.05 considered statistically significant. Data labelling and AI model development were conducted in a computer environment; labelled data were transferred to Excel and processed by the service provider using AI techniques.

## 3 Results

The study was completed with 1000 newborns. The mean gestational age of infants were 38.52 ± 1.07 weeks (min: 36 to max: 42) and postnatal age was 2.90 ± 0.77 days (min: 1 to max: 5). It was determined that all of infants were fed 1 h before the image was taken, did not use any painkillers/sedative medication, did not undergo surgical operation and did not have any serious health problems. Table 1 shows the descriptive characteristics of the newborns.

**Table 1:** Descriptive characteristics of newborns.

Variable	*n*	%
*Gender*		
Girl	638	63.8
Male	362	36.2
*Type of birth*		
Normal birth	643	64.3
Caesarean section	357	35.7
*Type of nutrition*		
Breast milk	895	89.5
Formula milk	59	5.9
Breast milk + formula milk	46	4.6
Total	1000	100.0

The mean N‐PASS scores evaluated by the three researchers were 4.62 ± 3.23, 4.60 ± 3.24, 4.63 ± 3.22, respectively. The agreement of the N‐PASS scores evaluated by the researchers was compared pairwise with Cohen's Kappa test. In pairwise comparisons, it was found that the agreement between Researcher 1 and Researcher 2 (Kappa Coefficient = 0.941; *p* < 0.001); Researcher 1 and Researcher 3 (Kappa Coefficient = 0.946; *p* < 0.001); and Researcher 2 and Researcher 3 (Kappa Coefficient = 0.909 *p* < 0.001) was high. The reason for looking at the agreement between the researchers is to reduce bias and increase the reliability of the data to be used in the creation of AI. Image and sound recordings of 1000 newborns evaluated by three different researchers and with high agreement between them were used in the creation of the AI model.

Out of 1000 newborns' images and voice recordings used in the creation of the AI model, 80% were used for training (800 newborns' images and voice recordings), 10% for validation (100 newborns' images and voice recordings), and 10% (100 newborns' images and voice recordings) for testing the model. The obtained model gave 82% successful results in the images allocated for testing.

## 4 Discussion

This study was conducted to develop an application to assess the pain levels of newborns with AI techniques. In the study, N‐PASS was transferred to the AI model and integrated into the mobile application. In this way, the most comprehensive pain scale used in newborns was transferred to the AI prediction model. This model was integrated into the mobile application, and it was aimed to increase the usability level. In the model created with AI techniques; it was found that the model had a high success rate (82%) in predicting the presence of pain correctly.

One of the parameters used in the detection of pain in infants is facial expression. In a study by Zamzmi et al. [19], the facial expressions of newborn infants were captured on camera and classified as having or not having pain. In a study by Brahnam et al. [20], the faces of newborns with gestational ages of 18−72 weeks were photographed, and pain prediction was performed by machine learning. In a study [21], a machine learning model created by classifying the facial expressions of infants was used in the pain detection of infants. Neonatal nurses classified the pain of infants with an analysis system in which the facial expressions of infants were captured on camera using the face coding system [22]. In the study by Giordano et al. [23], an AI system designed for automatic face recognition was used to detect pain in infants. This system was compared with nurses' pain assessments and found to give similar results [23]. In a study classifying the facial expressions of newborns in the NICU [24], it was determined that pain prediction was as successful as that of nurses. In the study by Carlini et al. [25], pain was detected by classifying the facial expressions of infants in the NICU and integrated into the mobile application. In a similar study [26], a classification model in which infants' pain is detected from facial expressions was integrated into a mobile application. An AI‐supported faces pain scale was integrated into the mobile application for the evaluation of postoperative pain in infants by parents at home [27]. In different studies [28, 29, 30, 31, 32, 33], pain in infants was detected by analysing facial expressions. Facial expressions are one of the behavioural indicators of pain, but may not be sufficient for pain assessment alone [34]. The use of a scale that includes the physiological indicators of infants along with other behavioural indicators may provide more concrete data about infants' pain. The present study is quite different from previous studies in this respect.

One of the parameters used in pain detection in infants is the crying sound. In a study [35], pain detection was performed by classifying the crying sound of infants. A prediction model was created for certain types of pain by interpreting the crying sounds of infants with a machine learning‐based model [36]. In a study conducted with the collaboration of engineers and nurses [37], pain detection was performed by analysing crying sounds in infants. The N‐PASS parameters used in our study also include infant is crying. The difference of the present study from the studies in the literature is that multiple behavioural responses and physiological responses are evaluated together instead of a single behavioural response. In this way, it is ensured that the pain estimation is more accurate.

Physical and bodily parameters are among the important parameters used in pain assessment in infants. In a study [38], it was determined that the accuracy rate of pain assessment made with models created with physical pain symptoms in infants was higher. It is stated that pain can be detected in the early period with continuous monitoring of facial and body movements of infants with AI‐supported applications [34].

In a study [39], brain activities of infants were examined by nurses in the assessment of infants' pain, and pain prediction was made. Nurses created a data set with physiological and behavioural data of newborns to be used in postoperative pain assessment, but pain assessment was not performed with AI [40]. In a similar study, a data set was created with the idea of making a pain assessment with the NIPS scale and transferring it to an AI model, but the model could not be created. It was suggested that a model using similar data should be created [41]. In the study by Cheng et al. [42], the pain of newborns was evaluated using the NIPS scale during blood sampling. It was determined that the nurse and AI model were successful in pain assessment, and their accuracy rates were compatible with each other [42]. In the literature [26, 35, 43, 44, 45], there are studies that evaluated separately by observing crying, facial expression, and behaviours in newborns. However, most of the studies are descriptive [25, 43, 46, 47]. The fact that some of the parameters used in the literature for pain assessment in newborns (crying, facial expression, movements) are also included in the scale we used shows that our study is in parallel with the literature. However, the N‐PASS, which we adapted to the AI model in our study, is superior to other scales in that it can be used in both acute and chronic pain, can be applied to both premature and term infants, and can be applied to infants on mechanical ventilators. Therefore, our study enabled rapid and safe assessment of many parameters, including pain (both behavioural and physiological parameters), on a single scale with AI techniques in all newborns.

The limitation of the study is that the study was conducted only with infants over 30 weeks of gestation.

## 5 Conclusions

It was found that the success rate of the created model in correctly predicting the presence of pain in newborns was high (82%). In order to increase the use of the AI model, it was transferred to a mobile application. Our suggestion is to extend the use of the developed AI model, especially in neonatal intensive care units. Our other suggestions are to transform different pain scales used in the field into a fast and practical model with AI techniques and to involve nurses more in multidisciplinary AI studies.

## Author Contributions

Ayfer Acikgoz conceived the study, designed the research framework, and supervised all stages of the study. She also contributed to defining the study variables, guiding the methodology, and critically reviewing all drafts of the manuscript. Deniz Yigit developed the study design, conducted the statistical analyses, interpreted the data in collaboration with the co‐authors, and took the lead in writing and drafting the manuscript. She also managed the reference organisation and ensured appropriate citation formatting. Ozer Celik contributed to the interpretation of the findings, supported the methodological structuring of the study, and assisted in drafting and refining the manuscript, particularly the Methods and Discussion sections.

## Ethics Statement

Prior to the start of the study, permission for using the scale and approvals for the study were obtained from the Eskişehir Osmangazi University Ethics Committee (Date: 09.05.2019/Decision No: 08) and the relevant hospital administration (05.10.2020). The parents were provided with information about the study in advance, and their written and verbal consent was obtained. Only those newborns whose parents consented were included in the procedures.

## Conflicts of Interest

The authors declare no conflicts of interest.

## Data Availability

The data that support the findings of this study are available from the corresponding author upon reasonable request.
